# Feasibility of active surveillance in patients with clinically T1b papillary thyroid carcinoma ≤1.5 cm in preoperative ultrasonography: MASTER study

**DOI:** 10.1530/ETJ-23-0258

**Published:** 2024-04-18

**Authors:** Sang-Hyeon Ju, Yong Bae Ji, Minchul Song, Joung Youl Lim, Da Beom Heo, Min-Gyu Kim, Jae Won Chang, Ho-Ryun Won, Yea Eun Kang, Eu Jeong Ku, Mijin Kim, Eun Kyung Lee, June Young Choi, Hyeong Won Yu, Young Joo Park, Jun-Ho Choe, Bon Seok Koo, the MASTER study group

**Affiliations:** 1 Department of Internal Medicine, Chungnam National University Hospital, Daejeon, Republic of Korea; 2 Department of Otolaryngology–Head and Neck Surgery, Hanyang University College of Medicine, Seoul, Republic of Korea; 3 Department of Otorhinolaryngology–Head and Neck Surgery, Chungnam National University Hospital, Daejeon, Republic of Korea; 4 Department of Otorhinolaryngology–Head and Neck Surgery, Chungnam National University College of Medicine, Daejeon, Republic of Korea; 5 Department of Otorhinolaryngology–Head and Neck Surgery, Chungnam National University Sejong Hospital, Sejong, Republic of Korea; 6 Department of Internal Medicine, Seoul National University Hospital Healthcare System Gangnam Center, Seoul, Republic of Korea; 7 Department of Internal Medicine, Pusan National University Hospital, Busan, Republic of Korea; 8 Department of Internal Medicine, Center for Thyroid Cancer, National Cancer Center, Goyang-si, Republic of Korea; 9 Department of Surgery, Seoul National University Bundang Hospital, Seongnam-si, Republic of Korea; 10 Department of Internal Medicine, Seoul National University College of Medicine, Seoul, Republic of Korea; 11 Division of Endocrine Surgery, Department of Surgery, Sungkyunkwan University School of Medicine, Suwon, Republic of Korea

**Keywords:** papillary thyroid carcinoma, active surveillance, tumor size, ultrasonography, occult lymph node metastasis

## Abstract

**Objective:**

Active surveillance (AS) is generally accepted as an alternative to immediate surgery for papillary thyroid carcinoma (PTC) measuring ≤1.0 cm (cT1a) without risk factors. This study investigated the clinicopathologic characteristics of PTCs measuring ≤2.0 cm without cervical lymph node metastasis (cT1N0) by tumor size group to assess the feasibility of AS for PTCs between 1.0 cm and 1.5 cm (cT1b^≤1.5^).

**Design:**

This study enrolled clinically T1N0 patients with preoperative ultrasonography information (*n*= 935) from a cohort of 1259 patients who underwent lobectomy and were finally diagnosed with PTC from June 2020 to March 2022.

**Results:**

The cT1b^≤1.5^ group (*n* = 171; 18.3 %) exhibited more lymphatic invasion and occult central lymph node (LN) metastasis with a higher metastatic LN ratio than the cT1a group (*n* = 719; 76.9 %). However, among patients aged 55 years or older, there were no significant differences in occult central LN metastasis and metastatic LN ratio between the cT1a, cT1b^≤1.5^, and cT1b^>1.5^ groups. Multivariate regression analyses revealed that occult central LN metastasis was associated with age, sex, tumor size, extrathyroidal extension, and lymphatic invasion in patients under 55, while in those aged 55 or older, it was associated only with age and lymphatic invasion.

**Conclusion:**

For PTC patients aged 55 years or older with cT1b^≤1.5^, AS could be a viable option due to the absence of a significant relationship between tumor size and occult central LN.

## Introduction

Thyroid cancer, which is the most common endocrine malignancy, has been increasing globally (1, 2). From 1990 to 2017, the annual age-standardized incidence rate (ASIR) of thyroid cancer worldwide increased from 2.11 to 3.15, with an annual increase of 1.59% (1). In South Korea, widespread screening for thyroid cancer led to a 15-fold increase in diagnoses from 1993 to 2011 (3). However, following this surge, the incidence then dropped to half of the peak by 2015 (4). Despite this reduction, South Korea still recorded the highest ASIR of 45.0 per 100,000 women in 2020, far surpassing the global average of 10.1 per 100,000 women (5). Contrastingly, mortality from thyroid cancer decreased by approximately 4.6–4.8% between 2003 and 2019 (4). Consequently, given the resulting prevalence of low-risk papillary thyroid carcinoma (PTC) patients and the burden posed by the potential complications, active surveillance (AS) has received increased attention (6, 7).

The current American Thyroid Association guideline suggests AS as an alternative to immediate surgery in patients with (a) very-low-risk tumors (T1a PTC without metastases, local invasion, and aggressive cytologic features), (b) high surgical risk, (c) short remaining life span, or (d) concurrent medical or surgical issues that need to be addressed first (8). A meta-analysis of AS trials found that tumor growth in maximum diameter by 3 mm or more was observed in 4.4% of low-risk PTC cases over an average follow-up period of 51.7 months (9). Previous studies aimed at identifying factors related to an increase in tumor size during AS have identified several risk factors, including younger age (10, 11), high serum thyroid-stimulating hormone levels (12), lack of calcification or presence of microcalcification in the tumor (13), and rich vascularity at the most recent follow-up (13). In addition to changes in tumor size, the detection of cervical lymph node (LN) metastases is another critical element of AS because cervical LN metastasis is associated with a higher recurrence rate (14, 15) and poorer survival rates in PTC patients, especially those aged ≥45 years (16). In patients with papillary thyroid microcarcinoma (PTMC) without palpable lymphadenopathy, microscopic central and lateral LN metastases were already present in 60.9% and 39.5% of cases, respectively (17). Newly detected metastatic cervical LN disease during AS is found in 1% of patients with PTMC and is thought to be caused by the progression of microscopic LN metastasis (9). The following indications are suggested for surgery following AS for PTMC: (1) the tumor diameter reaching 13 mm, (2) newly detected LN metastasis, (3) patient preference, and (4) the appearance of other thyroid or parathyroid diseases requiring surgery (18). Since newly detected LN metastasis triggers a shift in the treatment strategy to surgery, predicting macroscopic LN metastasis is important for determining whether AS is indicated. Therefore, we evaluated the risk of recurrence in metastatic lymph nodes removed from patients who could have been considered for AS, classifying the risk based on the extent and number of LN metastases as well as the presence of extra-nodal extension.

In recent studies, expanded inclusion criteria for AS, including PTCs smaller than 1.5 cm, have shown favorable results. No regional or distant metastases were reported, and only 3.5% and 3.8% of PTC cases exhibited tumor growth of 3 mm or more during 15 and 25 months of follow-up, respectively (19, 20). However, there is still no consensus on the tumor size threshold for AS. To expand the indications of AS, it is crucial to understand the detailed clinicopathologic characteristics, such as multifocality, minimal extrathyroidal extension, vascular invasion, lymphatic invasion, LN metastasis, and thyroiditis, of T1b PTC that are ≤1.5 cm. Therefore, in this study, we examined the clinicopathological characteristics of T1b PTC ≤1.5 cm and discussed the potential for AS based on these characteristics.

## Methods

### Study subjects

Eligible subjects for this study were selected from a cohort established for A Multicenter, Randomized, Controlled Trial for Assessing the Usefulness of Suppressing Thyroid Stimulating Hormone Target Levels after Thyroid Lobectomy in Low to Intermediate Risk Thyroid Cancer Patients (MASTER) study from June 2020 to March 2022 (21). The study was registered in the clinical research information system (CRIS) of Korea Centers for Disease Control and Prevention (KCT0004989) in May of 2020. The cohort inclusion criteria for the MASTER study were as follows: (1) age between 19 and 79 years old (male or nonpregnant female); (2) PTC patients who underwent lobectomy within 3 weeks as part of their treatment for PTC; (3) tumor size of ≤4 cm with no or minimal extrathyroidal extension (ETE), clinical stage N0 or N1a according to American Joint Committee on Cancer tumor, node, metastasis (TNM) 8th edition cancer staging (22); and (4) no nodule or if any nodules in the remaining lobes showing benign or low-suspicion ultrasonographic findings (Korean Thyroid Imaging Reporting and Data System (K-TIRADS) class 1–3 (23)) with Bethesda system category I–III by fine needle aspiration (24). This study was approved by the Ethics Committee of the Institutional Review Board (IRB) of each institution participating in the MASTER study (21). The institutions and corresponding IRB approval numbers are Seoul National University Hospital (H-1912-012-1084), Nowon Eulji Medical Center (EMCS 2020-04-020), Asan Medical Center (2020-0529), Inje University Busan Paik Hospital (19-0227), Chungnam National University Hospital (CNUH2020-03-091), Chungnam National University Sejong Hospital (CNUSH2020-11-018), Konyang University Hospital (KYUH2020-05-015), Daegu Catholic University Hospital (CR-20-125-L), Samsung Medical Center (SMC 2020-09-184), National Cancer Center (NCC 2020-0109), Seoul Metropolitan Government Seoul National University Boramae Medical Center (10-2020-035), Severance Hospital (2020-0202-001), Chungbuk National University Hospital (2019-11-012), Eulji University Hospital (2020-02-005), Dankook University Hospital (2019-11-033), Seoul National University Bundang Hospital (B-2006/618-403), Ewha Womans University Seoul Medical Center (SEUMC 2019-11-035), Hallym University Sacred Heart Hospital (HALLYM 2019-05-022), Pusan National University Hospital (H-2006-023-091), Seoul St. Mary’s Hospital (KC20EIDT0476), and the Gangnam Severance Hospital (2019-0794-001). All patients provided written informed consent for the extended use of their clinical data after receiving a full explanation of the purpose and nature of all procedures used in the MASTER study. In the MASTER study, 1269 patients were initially enrolled. Of these, the patients who met all the inclusion criteria, had postoperative confirmation of PTC (*n* = 1259), were classified as cN0 (*n* = 1239), had tumor size information in their ultrasonography reports (*n* = 963), and finally classified as cT1 (*n* = 935) were enrolled in this study. The final cohort of 935 patients was classified as either cT1a (*n* = 719) or cT1b (*n* = 216) as shown in Supplementary Fig. 1 (see section on supplementary materials given at the end of this article).

### Study design and aims

In this prospective cohort study, we compared subjects’ clinicopathological characteristics according to their preoperative tumor size.

The aims of this study were as follows:

1. To compare the clinicopathologic characteristics of patients with PTC less than 1.5 cm to those of patients with PTC less than 1.0 cm by age group.
2. To determine the factors associated with occult LN metastasis in patients with PTC by age group.

### Statistical analysis

**Figure 1:**
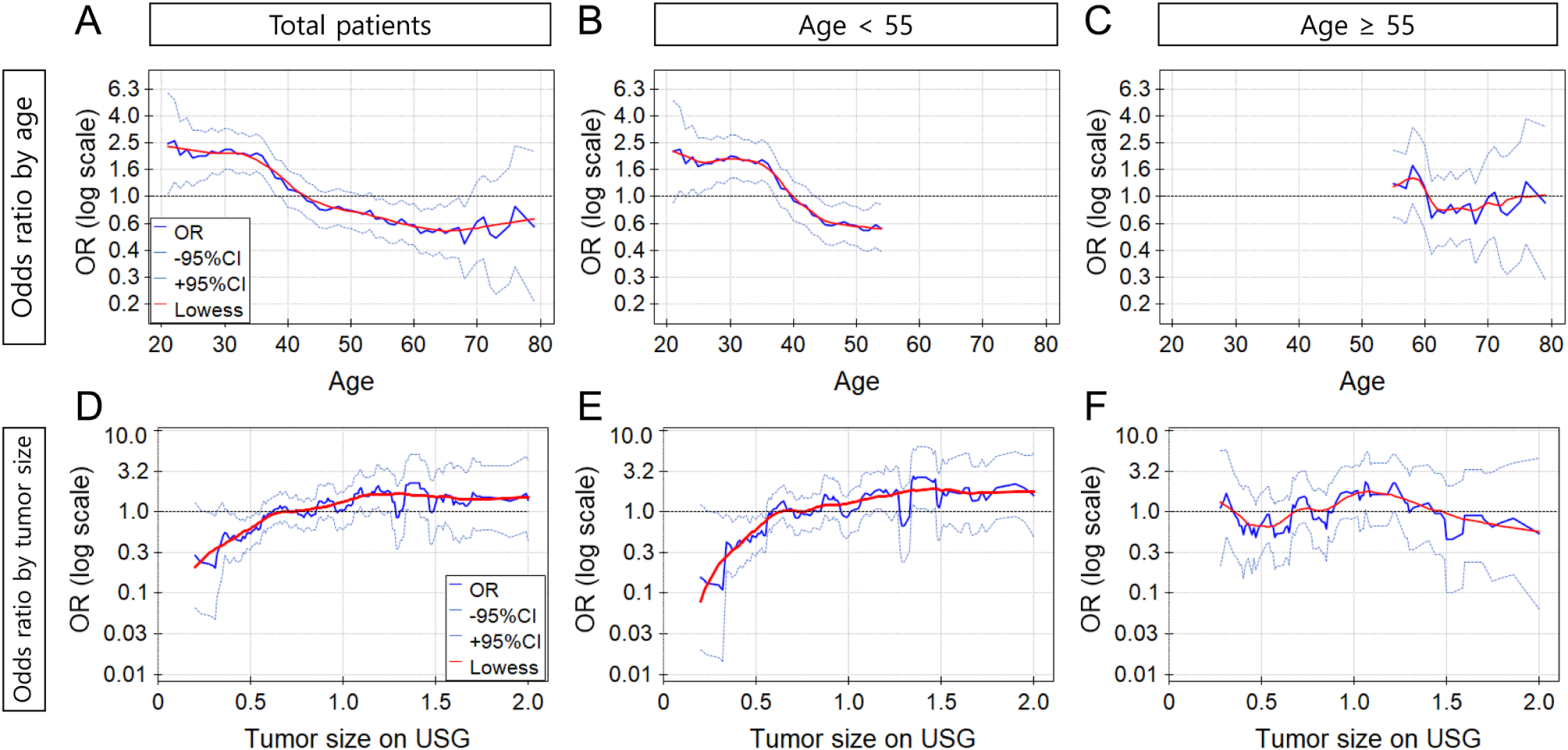
Odds ratio of occult central LN metastasis versus age or preoperative tumor size by age groups. (A–C) Odds ratio of occult central LN metastasis by age in total patents (A), age <55 (B), and age ≥55 (C). (D–F) Odds ratio of occult central LN metastasis by preoperative tumor size in total patients (D), age <55 (E), and age ≥55 (F). The solid red lines represent a smoothed and fitted relationship. The dashed lines represent odds ratio, and solid blue lines represent ±95% CI of odds ratio. Logistic regression analyses were used. LN, lymph node.

Numerical data are presented as the mean ± s.d ., while discrete data are shown as numbers along with their corresponding percentages (%). In order to compare the differences among numerical data from the three groups, a one-way analysis of variance (ANOVA) was conducted. Given the significance of the ANOVA results, *post hoc* pairwise comparisons were performed using the Bonferroni correction to control for type I error due to multiple comparisons. To compare the discrete data across the three groups, repeated chi-square tests were conducted. Given the significance of the results, *post hoc* adjustments were made using the Bonferroni correction to control for type I error due to multiple comparisons. Upon pairwise comparison of the three groups, the *P* value threshold was adjusted to 0.0167 using the Bonferroni correction. Statistical significance was determined based on this new threshold. We considered results statistically significant if *P* < 0.05 in numerical data and *P* < 0.0167 for discrete data. We utilized a generalized additive model with the PQstat version 1.8.6 (PQStat Software, Poznan, Poland) to visualize the nonlinear association between age and the odds ratio for occult central LN metastasis (Fig. 1). The data collection and analysis were conducted using SPSS Statistics version 26.0 (IBM Corp.) and GraphPad Prism 10.1.0 (GraphPad Software Inc.).

## Results

### Baseline characteristics

In the MASTER study, 1259 participants underwent lobectomy for low- to intermediate-risk PTC. Of these, 935 patients who were clinically diagnosed as cT1, measuring 2 cm or smaller in the greatest dimension without suspected extrathyroidal extension (ETE), and cN0 were finally included in this study (Supplementary Fig. 1, see section on supplementary materials given at the end of this article). The subjects’ baseline characteristics are summarized in Table 1.

**Table 1:** Demographic characteristics of the patients. Data are presented as mean ± s.d. or as *n* (%).

Characteristics	Values
*n*	935
Age, years	46.8 ± 11.6
BMI	24.8 ± 4.0
Height	162.4 ± 8.5
Body weight	65.9 ± 13.8
Sex	
Male	227 (24.3%)
Female	708 (75.7%)
Maximal tumor size, cm*	0.82 ± 0.34
Multifocality	
No	732 (78.3%)
Yes	203 (21.7%)
Minimal ETE	
No	534 (57.1%)
Yes	401 (42.9%)
Vascular invasion	
No	840 (89.8%)
Yes	95 (10.2%)
Lymphatic invasion	
No	595 (63.6%)
Yes	340 (36.4%)
Occult central LN metastasis	
No	653 (69.8%)
Yes	282 (30.2%)
AJCC 8th edition stage	
I	869 (92.9%)
II	66 (7.1%)
Hashimoto or lymphocytic thyroiditis	
No	690 (73.8%)
Yes	245 (26.2%)
Surgical procedure	
Lobectomy	935 (100.0%)
Central neck dissection	770 (82.4%)

*In preoperative USG.

AJCC, American Joint Committee on Cancer; BMI, body mass index; CND, central neck dissection; ETE, extrathyroidal extension; LN, lymph node; USG, ultrasonography.

### Clinicopathologic characteristics by tumor size on preoperative ultrasonography

The subjects were divided into three groups according to the largest preoperative dimension of the tumor on ultrasonography: cT1a (tumor size ≤ 1 cm), cT1b^≤1.5^ (1.0 < tumor ≤ 1.5 cm), and cT1b^>1.5^ (1.5 < tumor ≤ 2.0 cm) (Table 2). The cT1b^≤1.5^ group included a significantly higher proportion of men than the cT1a group. For numerical data, a one-way ANOVA was conducted followed by Bonferroni *post hoc* tests with *P* < 0.05 as the significance level; for discrete data, pairwise chi-square tests were used with significance adjusted to *P* < 0.0167 using the Bonferroni correction. On pathology results, lymphatic invasion and occult central LN metastasis were more common in the cT1b^≤1.5^ group than in the T1a group. In addition, the metastatic LN ratio, a prognostic factor related to disease-free survival that is calculated by dividing the number of metastatic LNs by the total number of dissected LNs (25), was higher in the cT1b^≤1.5^ group than in the T1a group. However, no significant differences were found in lymphatic invasion, occult central LN metastasis, and metastatic LN ratio between the cT1b^≤1.5^ and cT1b^>1.5^ groups. Multifocality, minimal ETE, vascular invasion, and thyroiditis showed no significant differences according to tumor size (Table 2).

**Table 2:** Clinicopathological characteristics of PTC patients based on tumor size. Data are presented as mean ± s.d. or as *n* (%). One-way ANOVA for numerical data followed by Bonferroni *post hoc* tests (*P* < 0.05 considered significant). Pairwise chi-square tests for discrete data, significance adjusted with Bonferroni correction (*P* < 0.0167 considered significant). Statistically significant *P* values are shown in bold.

	Tumor size, cm			*P*	
	A: ≤1.0	B: <1.0 to ≤1.5	C: <1.5 to ≤2.0	A vs B	B vs C
*n* (%)	719 (76.9 %)	171 (18.3 %)	45 (4.8 %)		
Age, years	46.9 ± 11.5	46.6 ± 11.8	46.3 ± 11.6	>0.999	>0.999
Sex (male, %)				**0.005**	0.860
Male	157 (21.8%)	55 (32.2%)	15 (33.3%)		
Female	562 (78.2%)	116 (67.8%)	30 (66.7%)		
Maximal tumor size (cm)	0.67 ± 0.18	1.22 ± 0.14	1.72 ± 0.15	**<0.001**	**<0.001**
Multifocality				0.754	0.045
No	566 (78.7%)	137 (80.1%)	29 (64.4%)		
Yes	153 (21.3%)	34 (19.9%)	16 (35.6%)		
Minimal ETE				0.071	0.503
No	426 (59.2%)	88 (51.5%)	20 (44.4%)		
Yes	293 (40.8%)	83 (48.5%)	25 (55.6%)		
Vascular invasion				0.020	0.255
No	659 (91.7%)	146 (85.4%)	35 (77.8%)		
Yes	60 (8.3%)	25 (14.6%)	10 (22.2%)		
Lymphatic invasion				**0.013**	0.181
No	479 (66.6%)	96 (56.1%)	20 (44.4%)		
Yes	240 (33.4%)	75 (43.9%)	25 (55.6%)		
Occult central LN metastasis				**<0.001**	0.867
No	526 (73.2%)	101 (59.1%)	26 (57.8%)		
Yes	193 (26.8%)	70 (40.9%)	19 (42.2%)		
Metastatic LN ratio*	0.15 ± 0.27	0.25 ± 0.34	0.29 ± 0.37	**<0.001**	>0.999
Hashimoto or lymphocytic thyroiditis				0.701	0.123
No	529 (73.6%)	123 (71.9%)	38 (84.4%)		
Yes	190 (26.4%)	48 (28.1%)	7 (15.6%)		

*The metastatic LN ratio is calculated by dividing the number of metastatic LNs by the total number of LNs dissected.

ETE, extrathyroidal extension; LN, lymph node.

Since younger age and positive LN metastasis are risk factors for progression during AS (26, 27, 28), we analyzed the clinicopathological characteristics in subpopulations divided by age. In patients under 55 years old (*n* = 669, 71.6% of the total patient cohort), similar to the findings from all subjects (Table 2), there were higher proportions of men and occult central LN metastasis, and the metastatic LN ratio was higher in the cT1b^≤1.5^ group than in the T1a group, while vascular and lymphatic invasion showed no significant difference between the two groups (Table 3). Between cT1b^≤1.5^ and cT1b^>1.5^ groups, there were no significant differences except tumor size (Table 3). However, in patients 55 or more years old (*n* = 266, 28.4% of the total patient cohort), occult central LN metastasis and the metastatic LN ratio were not different between the T1a and cT1b^≤1.5^ groups, but vascular invasion was significantly more common in cT1b^≤1.5^ compared to T1a (Table 4). Additionally, we repeated the analysis using a threshold of 45 years old and obtained results similar to those with a threshold of 55 years old. In patients under 45 years old (*n* = 396, 42.4% of the total patient cohort), the cT1b^≤1.5^ group demonstrated a greater prevalence of occult central LN metastasis and an elevated metastatic LN ratio compared to the cT1a group (Supplementary Table 1). In contrast, for patients aged 45 or older (*n* = 539, 57.6% of the total patient cohort), the cT1b^≤1.5^ group exhibited a higher prevalence of vascular and lymphatic invasion compared to the cT1a group (Supplementary Table 2). Additionally, the analysis of occult central LN metastasis between the cT1a and cT1b^≤1.5^ groups revealed marginally nonsignificant results (*P* = 0.024) in individuals aged 45 or older, as shown in Supplementary Table 2. However, this difference was clearly not significant (*P* = 0.249) in individuals aged 55 or older, according to Table 4. These differences in occult central LN metastasis driven by age cut-off of 45 and 55 are summarized in Supplementary Fig. 2. This observation suggests that a safer cutoff may be 55 years rather than 45 years old.

**Table 3:** Clinicopathological characteristics of PTC patients under 55 years old based on tumor size. Data are presented as mean ± s.d. or as *n* (%). One-way ANOVA for numerical data followed by Bonferroni *post hoc* tests (*P* < 0.05 considered significant). Pairwise chi-square tests for discrete data, significance adjusted with Bonferroni correction (*P* < 0.0167 considered significant). Statistically significant *P* values are shown in bold.

	Tumor size, cm			*P*	
	A: ≤1.0	B: <1.0 to ≤1.5	C: <1.5 to ≤2.0	A vs B	B vs C
*n* (%)	513 (76.7 %)	123 (18.4 %)	33 (4.9 %)		
Age, years	41.3 ± 8.4	40.9 ± 8.0	41.1 ± 8.3	>0.999	>0.999
Sex				**0.001**	>0.999
Male	115 (22.4%)	46 (37.4%)	12 (36.4%)		
Female	398 (77.6%)	77 (62.6%)	21 (63.6%)		
Maximal tumor size (cm)	0.67 ± 0.18	1.22 ± 0.14	1.72 ± 0.16	**<0.001**	**<0.001**
Multifocality				0.713	0.061
No	404 (78.8%)	99 (80.5%)	21 (63.6%)		
Yes	109 (21.2%)	24 (19.5%)	12 (36.4%)		
Minimal ETE				0.419	0.330
No	293 (57.1%)	65 (52.8%)	14 (42.4%)		
Yes	220 (42.9%)	58 (47.2%)	19 (57.6%)		
Vascular invasion				0.226	>0.999
No	469 (91.4%)	108 (87.8%)	29 (87.9%)		
Yes	44 (8.6%)	15 (12.2%)	4 (12.1%)		
Lymphatic invasion				0.066	0.330
No	318 (62%)	65 (52.8%)	14 (42.4%)		
Yes	195 (38%)	58 (47.2%)	19 (57.6%)		
Occult central LN metastasis				**0.001**	0.562
No	363 (70.8%)	67 (54.5%)	16 (48.5%)		
Yes	150 (29.2%)	56 (45.5%)	17 (51.5%)		
Metastatic LN ratio*	0.17 ± 0.28	0.29 ± 0.35	0.37 ± 0.39	**<0.001**	0.573
Hashimoto or lymphocytic thyroiditis				>0.999	0.251
No	377 (73.5%)	91 (74%)	28 (84.8%)		
Yes	136 (26.5%)	32 (26%)	5 (15.2%)		

*The metastatic LN ratio is calculated by dividing the number of metastatic LNs by the total number of LNs dissected.

ETE, extrathyroidal extension; LN, lymph node.

**Table 4:** Clinicopathological characteristics of PTC patients 55 or more years old based on tumor size. Data are presented as mean ± s.d. or as *n* (%). One-way ANOVA for numerical data followed by Bonferroni *post hoc* tests (*P* < 0.05 considered significant). Pairwise chi-square tests for discrete data, significance adjusted with Bonferroni correction (*P* < 0.0167 considered significant). Statistically significant *P* values are shown in bold.

	Tumor size, cm			*P*	
	A: ≤1.0	B: <1.0 to ≤1.5	C: <1.5 to ≤2.0	A vs B	B vs C
	206 (77.4 %)	48 (18.0 %)	12 (4.5 %)		
Age, years	60.7 ± 4.6	61.4 ± 4.9	60.8 ± 5.8	>0.999	>0.999
Sex				>0.999	0.692
Male	42 (20.4%)	9 (18.8%)	3 (25.0%)		
Female	164 (79.6%)	39 (81.3%)	9 (75.0%)		
Maximal tumor size (cm)	0.67 ± 0.18	1.21 ± 0.15	1.73 ± 0.16	**<0.001**	**<0.001**
Multifocality				>0.999	0.448
No	162 (78.6%)	38 (79.2%)	8 (66.7%)		
Yes	44 (21.4%)	10 (20.8%)	4 (33.3%)		
Minimal ETE				0.047	>0.999
No	133 (64.6%)	23 (47.9%)	6 (50.0%)		
Yes	73 (35.4%)	25 (52.1%)	6 (50.0%)		
Vascular invasion				**0.014**	0.066
No	190 (92.2%)	38 (79.2%)	6 (50.0%)		
Yes	16 (7.8%)	10 (20.8%)	6 (50.0%)		
Lymphatic invasion				0.062	0.508
No	161 (78.2%)	31 (64.6%)	6 (50.0%)		
Yes	45 (21.8%)	17 (35.4%)	6 (50.0%)		
Occult central LN metastasis				0.249	0.486
No	163 (79.1%)	34 (70.8%)	10 (83.3%)		
Yes	43 (20.9%)	14 (29.2%)	2 (16.7%)		
Metastatic LN ratio*	0.11 ± 0.23	0.18 ± 0.30	0.06 ± 0.16	0.411	0.589
Hashimoto or lymphocytic thyroiditis				0.370	0.317
No	152 (73.8%)	32 (66.7%)	10 (83.3%)		
Yes	54 (26.2%)	16 (33.3%)	2 (16.7%)		

*The metastatic LN ratio is calculated by dividing the number of metastatic LNs by the total number of LNs dissected.

ETE, extrathyroidal extension; LN, lymph node.

To predict the clinical outcomes by detailed characteristics of LNs, we categorized all the occult central LN metastases cases (*n* = 282, Table 1) in low risk N1 (cN0, micro-metastasis, small LN metastasis, ≤5 small LN metastases, and no extra-nodal extension) and high risk N1 (one or more of cN1, metastatic LN >3cm, >5 metastatic LNs, or extra-nodal extension) (29). The proportion of high risk N1 did not significantly differ between tumor size groups, both in the total patient cohort and in age subgroups divided by ages of 55 and 45 (Supplementary Table 3). Importantly, the proportion of high risk N1 in the younger age group was significantly higher than in the older age group; 33 patients (14.8 %) had high risk N1 in age <55, while 2 patients (3.3 %) had high risk N1 in age ≥55 (*P* = 0.015). Similarly, 26 patients (17.3 %) had high risk N1 in age <45, while 9 patients (6.8 %) had high risk N1 in age ≥45 (*P* = 0.010) (Supplementary Table 3). Taken together, our findings reveal that younger patients in our cohort are more likely to have high-risk N1 disease.

### Clinicopathologic factors associated with occult central LN metastasis

We found several factors associated with occult central LN metastasis from logistic regression analysis. In total patients, multivariate analyses identified age, tumor size, minimal ETE, and lymphatic invasion as factors influencing occult central LN metastasis (Supplementary Table 4). Similarly, in patients under 55 years old, age, sex, tumor size, minimal ETE, and lymphatic invasion were identified as factors associated with occult central LN metastasis (Supplementary Table 5). In contrast, occult central LN metastasis in patients 55 or more years old was associated with only age and lymphatic invasion in the multivariate analysis (Table 5). A nonlinear association between patients’ age and the log-transformed odds ratio of occult central LN metastasis was observed using local regression smoothing in a generalized additive model (Fig. 1). In total patients, and specifically in those under 55 years old, an increase in age was negatively associated with occult central LN metastasis (Fig. 1A and B). However, this negative association was not consistently observed in patients aged 55 or older (Fig. 1C). However, tumor size showed a positive association with the odds ratio for occult LN metastasis in all patients and those under 55 years old, showing a plateau in patients with a tumor size greater than 1.5 cm (Fig. 1D and E). Importantly, in patients aged 55 or older, a positive association between tumor size and occult central LN metastasis was not found (Fig. 1F). Collectively, these results suggest that tumor size influences the presence of occult central LN metastasis differently by age group.

**Table 5:** Clinicopathologic factors associated with occult central LN metastasis in patients aged 55 years and older. Univariate and multivariate logistic regression. Values in bold indicate statistical significance (*P* < 0.05).

Parameters	Univariate regression		Multivariate regression	
	*P*	Odds ratio (95% CI)	*P*	Odds ratio (95% CI)
Age	0.152	0.95 (0.89–1.02)	**0.008**	0.83 (0.73–0.95)
Sex (ref.: female)	0.068	1.86 (0.96–3.62)	0.636	0.77 (0.25–2.31)
Tumor size	0.449	1.38 (0.60–3.20)	0.973	1.02 (0.25–4.13)
Multifocality	0.264	1.46 (0.75–2.85)	0.328	1.62 (0.62–4.27)
Minimal ETE	0.037	1.86 (1.04–3.33)	0.457	1.42 (0.56–3.61)
Vascular invasion	0.081	2.03 (0.92–4.50)	0.402	0.49 (0.09–2.62)
Lymphatic invasion	<0.001	4.60 (2.48–8.55)	**<0.001**	7.09 (2.67–18.81)
Thyroiditis	0.992	1.00 (0.52–1.92)	0.128	2.11 (0.81–5.51)
*BRAF* mutation	0.438	0.67 (0.24–1.87)	0.348	0.57 (0.17–1.85)

ETE, extrathyroidal extension; LN, lymph node; ref., reference.

## Discussion

The primary aim of this study was to investigate the clinicopathological characteristics of T1b PTC that is smaller than 1.5 cm (T1b^≤1.5^), with the goal of providing insight into the feasibility of AS as an alternative to immediate surgery in these cases. Notably, cT1b^≤1.5^ cases, when compared to T1a, had more aggressive features, such as higher frequencies of occult central LN metastasis, as well as a higher metastatic LN ratio. However, this pattern was primarily observed in patients under the age of 55. In patients aged 55 years and older, the clinical characteristics of cT1b^≤1.5^ were similar to those of T1a, with the exception of vascular invasion. This observation suggests that AS could be a reasonable management strategy for patients over 55 years of age with cT1b^≤1.5^. Multivariate regression analysis demonstrated that the presence of occult central LN metastasis in patients under 55 years old was associated with multiple factors, including age, sex, tumor size, minimal ETE, and lymphatic invasion. However, in patients aged 55 years or older, it was associated only with age and lymphatic invasion.

Since the first clinical application of AS for patients with low-risk PTMC in 1993, numerous clinical trials have been conducted to assess its safety and efficacy (30). While most of these studies focused on low-risk PTMC, some have broadened their scope to include T1b PTC. A comparison of clinicopathologic characteristics between T1a and T1b in the MASTER cohort revealed significant differences in minimal ETE, LVI, occult central LN metastasis, and metastatic LN ratio. Our results align with these findings, with the comparison in patients over 55 years of age showing a difference only in minimal ETE, which supports the potential for AS in T1b as well as T1a (31). In 2017, a prospective study of AS in PTC ≤1.5 cm included 59 patients (20.3% of the total 291 patients enrolled) with PTC measuring 1.1−1.5 cm (19). Of these, only two out of 59 patients (3.4 %) exhibited an increase in tumor size of 3 mm or more, a rate even lower than that of patients with PTMC (9/232; 3.9%). Moreover, no regional or distant metastases developed during AS (19). In 2018, another prospective study of AS in PTC <1.5 cm reported that 21 out of 57 patients (36.9%) had T1b PTC, and only two patients (3.5%) showed tumor growth of more than 3 mm during the median follow-up of 13.3 months (20). In 2019, a prospective trial from Japan reported an average of 7.4 years of AS results from 61 PTC patients with T1bN0M0 stage (32). Four patients (7%) showed tumor growth, and development of LN metastasis was observed in two (3%) out of 61 T1b PTC patients. No significant difference in tumor growth and LN metastasis between T1a and T1b was found. The authors identified weak calcification and rich vascularity as risk factors for tumor growth (32). In 2022, a prospective nonrandomized controlled trial enrolled 87 patients with 10.1−15.0 mm PTC and 46 patients with 15.1−20.0 mm PTC (33). Between T1a and T1b PTC, there was no significant difference in size growth of more than 3 mm or 5 mm. No patients developed metastatic LNs or distant metastasis during a mean 37.1 months of follow-up (33). Additionally, they found that the initial size of the tumor was not associated with tumor growth or volume growth, which has also been reported in a meta-analysis (28, 33). To date, several prospective studies of AS on T1b PTC have resulted in favorable clinical outcomes. However, to identify tumor size thresholds for safe and effective AS for PTC, large-scale, well-structured studies are required.

Older age is a well-known prognostic factor of PTC. Seminal studies on the prognosis of thyroid cancer have established prognostic systems, in which patient age is an essential component (22, 34, 35, 36). Based on these studies, expanding the indications of AS up to 1.5 cm for individuals aged 55 or older might seem counterintuitive, but the unique characteristics of our cohort should be taken into consideration. A study using the Surveillance, Epidemiology, and End Results (SEER) population-based database found that an increase in patient age was associated with a higher proportion of male patients, poorly differentiated or undifferentiated pathologic grades, and the presence of distant metastasis (i.e. more advanced TNM stage) (37). Therefore, it is important to note that our cohort only included cT1N0 PTC patients, regardless of their age. This could potentially exclude older patients with a poorer prognosis, resulting in seemingly more favorable clinicopathologic characteristics among the older PTC patients. A SEER-based cohort study demonstrated a U-shaped relationship between age and LN metastasis rate, with the lowest rate in the middle age range of 40−60 years (38, 39). In our cohort, the risk of occult central LN metastasis decreased with age up to 60−70 years old. The difference between the SEER cohort and our cohort is that the latter demonstrated a blunted risk of LN metastasis in patients older than 60 years, resulting in an older nadir age than in the SEER study (Fig. 1A). These differences could be attributed to the inclusion criteria of our cohort, which was limited to only cT1N0 PTC. Moreover, the risk of recurrence, determined by the extent and number of metastatic LNs and extra-nodal extension, was more favorable in older age (Supplementary Table 3). A higher proportion of low-risk N1 disease is expected to play a role in the slow progression of the disease in older age (26). For PTC, because not all nodes are the same, categorizing these nodes by risk level will aid in tailoring the individualization of AS strategy.

In our study, we adopted an age cut-off of 55 years based on the clinicopathologic characteristics, esp. LN metastasis. In a retrospective study on AS in PTMC, the authors suggested risk factors of tumor enlargement as young age (<40 years), large tumor size (≥9 mm), and a high detailed TSH score (higher than the lower normal limit) (40). They selected the age cut-off of 40 years based on the difference in tumor enlargement rates of PTMC between age groups, and novel LN metastasis was also associated with younger age (<40 years) according to multivariate analysis. There are several differences between their study and ours: they conducted a retrospective observational study, whereas ours is a cross-sectional study; they enrolled patients with PTMC, but we enrolled patients with both cT1a and cT1b and focused on cT1b^≤1.5^; and regarding the focus of the study, they tracked tumor enlargement and the appearance of novel LN metastasis, while our study aimed to evaluate the feasibility of cT1b^≤1.5^ for AS based on evaluating occult central LN metastasis (40). These differences could attribute to the difference in the age cut-offs. In a previous study, younger PTC patients showed a higher LN metastasis rate but a lower mortality rate. A study using a SEER-based cohort concluded that cervical LN metastasis was an independent risk factor for overall survival only in PTC patients over 45 years old, but not in those under 45 years old (16). In another retrospective study, the postoperative LN recurrence and distant recurrence rates were higher in PTC patients younger than 20 years and older than 60 years of age, but the carcinoma death rate was lower at a younger age and gradually increased with age (41). The specific reasons why younger PTC patients have better outcomes, despite an increased risk of LN metastasis, remain unclear. Potential explanations could include spontaneous resolution of LN metastasis (38), better general health and anti-tumor immunity, and fewer comorbidities in younger individuals, all of which might contribute to the lower mortality rate from PTC. Further studies are needed to understand the natural course of LN metastasis in PTC.

Angiolymphatic invasion is composed of two distinct invasions, vascular and lymphatic invasions, that possibly have an impact on the prognosis of PTC. The vascular invasion in PTC has been proposed as a prognostic factor of distant metastasis at diagnosis and a higher incidence of recurrence (42, 43). However, a study in well-differentiated PTC showed that the presence of vascular invasion is not an independent predictor of the outcome based on their multivariate analysis (44). Though vascular invasion is considered to have an association with prognosis, in the context of considering multiple factors, the significance is outweighed by central LN metastasis (45, 46). Despite vascular invasion’s inferiority in prognostication, a study reported a positive relationship between lymphovascular invasion and PTC tumor size, suggesting a tumor size of 1.5 cm as a safe upper limit for AS (47). In the case of lymphatic invasion, though the lymphatic invasion itself is not included as a member of risk stratification, several studies suggest its independent prognostic significance. Patients with microscopic lymphatic invasion were associated with a larger primary tumor, multifocality, LN metastases (48), and persistent/recurrent disease (48, 49). In another study that enrolled 3381 PTC patients, vascular invasion, not lymphatic invasion, was associated with locoregional recurrence (50). Collectively, until now, vascular invasion rather than lymphatic invasion is used for risk stratification. In our study, lymphatic invasion was a parameter associated with occult central LN metastasis in the total patient group and subgroups divided by the age of 55 (Supplementary Tables 4, 5 and Table 5). Given the consistent association of lymphatic invasion with LN metastasis, further studies that reinforce the evidence for the prognostic value of lymphatic invasion are required.

This study has several strengths that underscore its contribution to the existing body of knowledge concerning AS in low-risk PTC management. First, our analysis is supported by a large sample size (*n* = 935) derived from the MASTER cohort, a multicenter, randomized, controlled trial. The multicentric nature of our data also strengthens the external validity of our findings, allowing broader generalizability. Secondly, sonographic tumor sizes were utilized for classification by tumor size. Ultrasound measurements of tumor size are typically 10% larger than pathologic measurements (51), and sonographic tumor sizes are more practical for clinical use. Thirdly, although the patients underwent lobectomy for cT1N0M0 PTC rather than adopting AS, their clinicopathologic profile could potentially represent Korean candidates for AS. In this context, our data serve as a direct source of insight into the initial pathological status of these prospective AS candidates. However, the study may have certain limitations. First, because the MASTER study was not designed for AS, this cross-sectional study can only provide initial pathology information, but no longitudinal outcomes. The long-term implications of AS for cT1b^≤1.5^ PTC should be validated through prospective long-term follow-up study. Secondly, potential selection bias could exist. The study only included patients from the MASTER cohort, a Korean cohort, who were eligible for lobectomy. Thirdly, more detailed genetic information, including mutation profiles beyond *BRAF* and *RAS* and transcriptomic analyses that could offer additional implications, was not investigated (52). Further prospective studies that include a more diverse cohort of patients undergoing AS, as well as incorporating genetic profiling, could help overcome these limitations and provide more comprehensive insights.

In conclusion, our study evaluated the feasibility of AS as an alternative to immediate surgery for cT1b^≤1.5^ PTC based on the initial sonographic tumor size and the clinicopathologic profile. The findings suggest that while cT1b^≤1.5^ PTC presented more aggressive features than T1a PTC, patients aged 55 or older with cT1b^≤1.5^ PTC showed similar characteristics to those with T1a PTC. Therefore, AS may be a feasible approach for cT1b^≤1.5^ PTC patients aged 55 or older.

## Supplementary Materials

Supplementary Figure S1. Patient inclusion process

Supplementary Figure S2. Proportion of occult central lymph node metastasis by tumor size and age group of 45 or 55

Supplementary Table S1. clinicopathological characteristics of PTC patients under 45 years old based on tumor size

Supplementary Table S2. clinicopathological characteristics of PTC patients 45 or more years old based on tumor size

Supplementary Table S3. Risk of recurrence classification of lymph node metastasis by age and tumor size

Supplementary Table S3. Risk of recurrence classification of lymph node metastasis by age and tumor size

Supplementary Table S5. Clinicopathologic factors associated with occult central LN metastasis in patients younger than 55 years

## Declaration of interest

The authors declare that there is no conflict of interest that could be perceived as prejudicing the impartiality of the study reported.

## Funding

This research was supported by a grant from the Patient-Centered Clinical Research Coordinating Center (PACEN) funded by the Ministry of Healthhttp://dx.doi.org/10.13039/100009647 & Welfare, Republic of Korea (grant number: HC19C0103).

## Data availability

The datasets generated during and/or analyzed during the current study are available from the corresponding author on reasonable request.

## Author contribution statement

S-HJ and YBJ performed formal analysis and wrote the original draft. S-HJ visualized the results. MS validated the results. YBJ, MS, JYL, DBH, M-GK, JWC, H-RW, YEK, EJK, MK, EKL, JYC, HWY, and J-HC conducted investigations. YJP and BSK conceived and designed the study. YJP acquired funding. J-HC and BSK reviewed and edited the writing and they supervised the study process. All authors contributed to the article and approved the submitted version.
