# Supplementary material for: Feasibility of active surveillance in patients with clinically T1b papillary thyroid carcinoma ≤1.5 cm in preoperative ultrasonography: MASTER study
Source: Eur Thyroid J. 2024 Apr 18;13(2):e230258. doi: 10.1530/ETJ-23-0258 (PMC11046321; doi:10.1530/ETJ-23-0258)

Supplementary Figure S1. Patient inclusion process

From the MASTER study cohort, we selected participants who had preoperative ultrasonography data, were classified as cN0 and cT1, and were confirmed as papillary thyroid carcinoma post-operatively (N=935). PTC, papillary thyroid carcinoma; USG, ultrasonography.

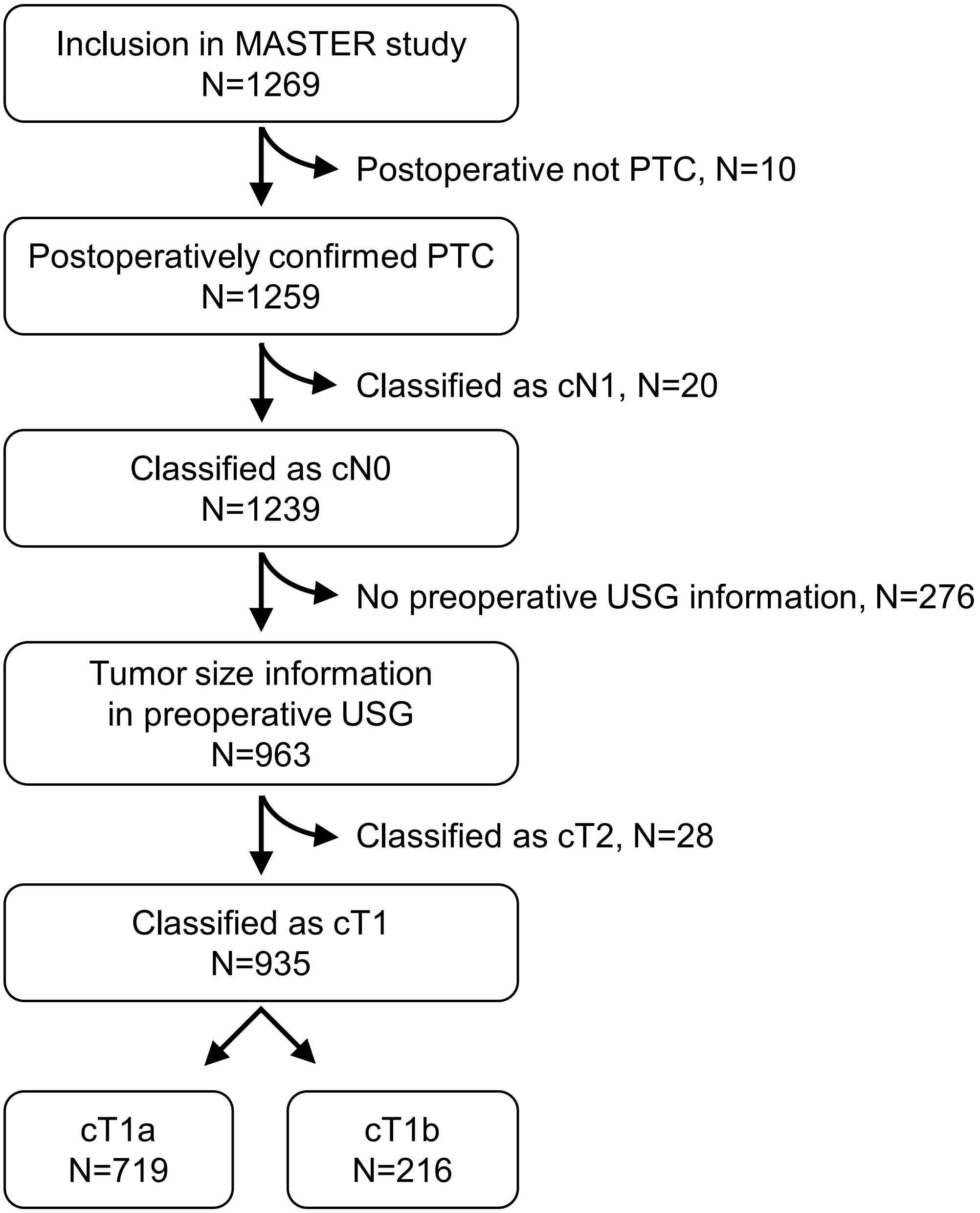

Supplement: Supplementary Figure S1. Patient inclusion process [file supplementary_figure_1.pdf]
