# Supplementary material for: Feasibility of active surveillance in patients with clinically T1b papillary thyroid carcinoma ≤1.5 cm in preoperative ultrasonography: MASTER study
Source: Eur Thyroid J. 2024 Apr 18;13(2):e230258. doi: 10.1530/ETJ-23-0258 (PMC11046321; doi:10.1530/ETJ-23-0258)
Supplement: Supplementary Figure S2. Proportion of occult central lymph node metastasis by tumor size and age group of 45 or 55 [file supplementary_figure_2.pdf]

Supplementary Figure S2. Proportion of occult central lymph node metastasis by tumor size and age group of 45 or 55

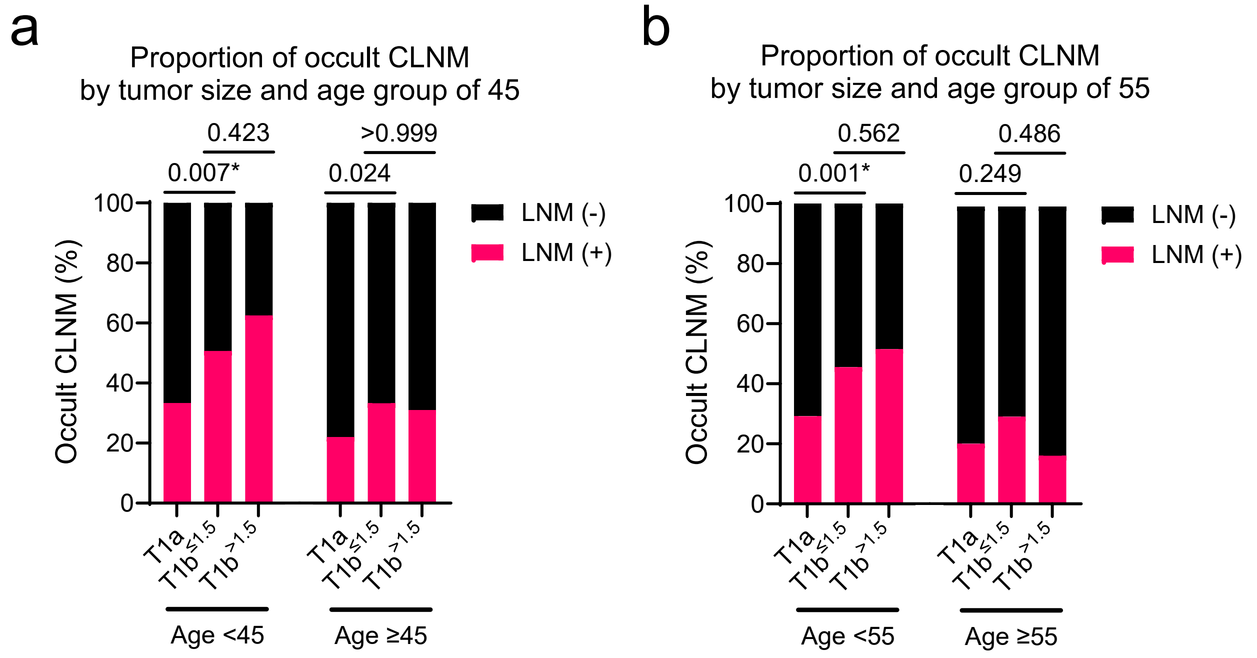

(a) Proportion of occult CLNM by tumor size group (cT1a, cT1b$^{\leq 1.5}$, and cT1b$^{>1.5}$) in age <45 and ≥45 group. (b) Proportion of occult CLNM by tumor size group (cT1a, cT1b$^{\leq 1.5}$, and cT1b$^{>1.5}$) in age <55 and ≥55 group. CLNM, central lymph node metastasis. Pairwise Chi-square tests were used and the statistical significance was adjusted with Bonferroni correction (*$p < 0.0167$ considered significant).
