# Supplementary material for: Feasibility of active surveillance in patients with clinically T1b papillary thyroid carcinoma ≤1.5 cm in preoperative ultrasonography: MASTER study
Source: Eur Thyroid J. 2024 Apr 18;13(2):e230258. doi: 10.1530/ETJ-23-0258 (PMC11046321; doi:10.1530/ETJ-23-0258)
Supplement: Supplementary Table S1. clinicopathological characteristics of PTC patients under 45 years old based on tumor size [file supplementary_table_1.pdf]

Supplementary Table S1. clinicopathological characteristics of PTC patients under 45 years old based on tumor size

	①Tumor ≤ 1.0 cm N=305 (77.0 %)	②1.0<Tumor ≤ 1.5 cm N=75 (18.9 %)	③1.5<Tumor ≤ 2.0 cm N=16 (4.0 %)	*p* value ①vs②	*p* value ②vs③
Age, years	35.8 ± 6.0	35.5 ± 5.2	33.6 ± 4.6	>0.999	0.645
Sex (male, %)				0.022	>0.999
Male	79 (25.9%)	30 (40.0%)	6 (37.5%)		
Female	226 (74.1%)	45 (60.0%)	10 (62.5%)		
Maximal tumor size (cm)	0.67 ± 0.18	1.22 ± 0.14	1.72 ± 0.18	**<0.001**	**<0.001**
Multifocality				>0.999	0.092
No	250 (82.0%)	62 (82.7%)	10 (62.5%)		
Yes	55 (18.0%)	13 (17.3%)	6 (37.5%)		
Minimal ETE				0.796	0.270
No	176 (57.7%)	42 (56.0%)	6 (37.5%)		
Yes	129 (42.3%)	33 (44.0%)	10 (62.5%)		
Vascular invasion				0.825	0.586
No	276 (90.5%)	69 (92.0%)	16 (100%)		
Yes	29 (9.5%)	6 (8.0%)	0		
Lymphatic invasion				0.897	0.597
No	178 (58.4%)	43 (57.3%)	8 (50.0%)		
Yes	127 (41.6%)	32 (42.7%)	8 (50.0%)		
Occult central LN metastasis				**0.007**	0.423
No	203 (66.6%)	37 (49.3%)	6 (37.5%)		
Yes	102 (33.4%)	38 (50.7%)	10 (62.5%)		
Metastatic LN ratio*	0.21 ± 0.31	0.36 ± 0.38	0.47 ± 0.41	**0.003**	0.800
Hashimoto or lymphocytic thyroiditis				0.761	0.730
No	232 (76.1%)	59 (78.7%)	14 (87.5%)		
Yes	73 (23.9%)	16 (21.3%)	2 (12.5%)		

Data are presented as mean ± S.D. or number (corresponding percentage). One-way ANOVA for numerical data followed by Bonferroni post-hoc tests ($p < 0.05$ considered significant). Pairwise Chi-square tests for discrete data, significance adjusted with Bonferroni correction ($p < 0.0167$ considered significant). Statistically significant *p* values are shown in bold. *The metastatic LN ratio is calculated by dividing the number of metastatic LNs by the total number of LNs dissected. ETE, extrathyroidal extension; LN, lymph node.
