# Supplementary material for: Feasibility of active surveillance in patients with clinically T1b papillary thyroid carcinoma ≤1.5 cm in preoperative ultrasonography: MASTER study
Source: Eur Thyroid J. 2024 Apr 18;13(2):e230258. doi: 10.1530/ETJ-23-0258 (PMC11046321; doi:10.1530/ETJ-23-0258)
Supplement: Supplementary Table S2. clinicopathological characteristics of PTC patients 45 or more years old based on tumor size [file supplementary_table_2.pdf]

Supplementary Table S2. clinicopathological characteristics of PTC patients 45 or more years old based on tumor size

	①Tumor ≤ 1.0 cm N=414 (76.8 %)	②1.0<Tumor ≤ 1.5 cm N=96 (17.8 %)	③1.5<Tumor ≤ 2.0 cm N=29 (5.4 %)	*p* value ①vs②	*p* value ②vs③
Age, years	55.1 ± 6.8	55.3 ± 7.3	53.4 ± 7.6	>0.999	0.571
Sex (male, %)				0.122	0.637
Male	78 (18.8%)	25 (26.0%)	9 (31.0%)		
Female	336 (81.2%)	71 (74.0%)	20 (69.0%)		
Maximal tumor size (cm)	0.66 ± 0.19	1.22 ± 0.14	1.73 ± 0.14	**<0.001**	**<0.001**
Multifocality				0.789	0.219
No	316 (76.3%)	75 (78.1%)	19 (65.5%)		
Yes	98 (23.7%)	21 (21.9%)	10 (34.5%)		
Minimal ETE				0.029	>0.999
No	250 (60.4%)	46 (47.9%)	14 (48.3%)		
Yes	164 (39.6%)	50 (52.1%)	15 (51.7%)		
Vascular invasion				**0.001**	0.132
No	383 (92.5%)	77 (80.2%)	19 (65.5%)		
Yes	31 (7.5%)	19 (19.8%)	10 (34.5%)		
Lymphatic invasion				**0.001**	0.210
No	301 (72.7%)	53 (55.2%)	12 (41.4%)		
Yes	113 (27.3%)	43 (44.8%)	17 (58.6%)		
Occult central LN metastasis				0.024	>0.999
No	323 (78.0%)	64 (66.7%)	20 (69.0%)		
Yes	91 (22.0%)	32 (33.3%)	9 (31.0%)		
Metastatic LN ratio*	0.11 ± 0.23	0.17 ± 0.28	0.17 ± 0.29	0.094	>0.999
Hashimoto or lymphocytic thyroiditis				0.322	0.110
No	297 (71.7%)	64 (66.7%)	24 (82.8%)		
Yes	117 (28.3%)	32 (33.3%)	5 (17.2%)		

Data are presented as mean ± S.D. or number (corresponding percentage). One-way ANOVA for numerical data followed by Bonferroni post-hoc tests ($p < 0.05$ considered significant). Pairwise Chi-square tests for discrete data, significance adjusted with Bonferroni correction ($p < 0.0167$ considered significant). Statistically significant *p* values are shown in bold. *The metastatic LN ratio is calculated by dividing the number of metastatic LNs by the total number of LNs dissected. ETE, extrathyroidal extension; LN, lymph node.
