# Supplementary material for: Feasibility of active surveillance in patients with clinically T1b papillary thyroid carcinoma ≤1.5 cm in preoperative ultrasonography: MASTER study
Source: Eur Thyroid J. 2024 Apr 18;13(2):e230258. doi: 10.1530/ETJ-23-0258 (PMC11046321; doi:10.1530/ETJ-23-0258)
Supplement: Supplementary Table S3. Risk of recurrence classification of lymph node metastasis by age and tumor size [file supplementary_table_3.pdf]

Supplementary Table S4. Clinicopathologic factors associated with occult central LN metastasis in total patients

Parameters	Univariate regression		Multivariate regression	
	p value	Odds ratio (95% CI)	*p* value	Odds ratio (95% CI)
Age	<0.001	0.97 (0.95–0.98)	**<0.001**	**0.96 (0.95–0.98)**
Sex (ref.: female)	<0.001	1.81 (1.32–2.48)	0.060	1.52 (0.98–2.34)
Tumor size	<0.001	2.55 (1.71–3.81)	**0.005**	**2.17 (1.26–3.73)**
Multifocality	0.409	1.15 (0.82–1.61)	0.220	1.31 (0.85–2.02)
Minimal ETE	<0.001	2.02 (1.52–2.68)	**0.003**	**1.82 (1.23–2.69)**
Vascular invasion	0.029	1.63 (1.05–2.52)	0.142	0.50 (0.20–1.26)
Lymphatic invasion	<0.001	4.51 (3.36–6.07)	**<0.001**	**3.48 (2.37–5.11)**
Thyroiditis	0.885	0.98 (0.71–1.34)	0.333	1.23 (0.81–1.89)
BRAF mutation	0.608	1.15 (0.68–1.95)	0.524	0.83 (0.46–1.49)

Ref., reference; ETE, extrathyroidal extension; LN, lymph node. Univariate and multivariate logistic regression.
