# Supplementary material for: Feasibility of active surveillance in patients with clinically T1b papillary thyroid carcinoma ≤1.5 cm in preoperative ultrasonography: MASTER study
Source: Eur Thyroid J. 2024 Apr 18;13(2):e230258. doi: 10.1530/ETJ-23-0258 (PMC11046321; doi:10.1530/ETJ-23-0258)
Supplement: Supplementary Table S3. Risk of recurrence classification of lymph node metastasis by age and tumor size [file supplementary_table_4.pdf]

Supplementary Table S3. Risk of recurrence classification of lymph node metastasis by age and tumor size

**In total patients**	①Tumor ≤ 1.0 cm N=193 (68.4 %)	②1.0<Tumor ≤ 1.5 cm N=70 (24.8 %)	③1.5<Tumor ≤ 2.0 cm N=19 (6.7 %)	*p* value ①vs②	*p* value ②vs③
Low risk N1 (N=247)	174 (90.2 %)	57 (81.4 %)	16 (84.2 %)	0.085	>0.999
High risk N1 (N=35)	19 (9.8 %)	13 (18.6 %)	3 (15.8 %)		
**In Age <55**	①Tumor ≤ 1.0 cm N=150 (67.3 %)	②1.0<Tumor ≤ 1.5 cm N=56 (25.1 %)	③1.5<Tumor ≤ 2.0 cm N=17 (7.6 %)	*p* value ①vs②	*p* value ②vs③
Low risk N1 (N=190)	132 (88.0 %)	44 (78.6 %)	14 (82.4 %)	0.119	>0.999
High risk N1 (N=33)	18 (12.0 %)	12 (21.4 %)	3 (17.6 %)		
**In Age ≥55**	①Tumor ≤ 1.0 cm N=43 (72.9 %)	②1.0<Tumor ≤ 1.5 cm N=14 (23.7 %)	③1.5<Tumor ≤ 2.0 cm N=2 (3.4 %)	*p* value ①vs②	*p* value ②vs③
Low risk N1 (N=57)	42 (97.7 %)	13 (92.9 %)	2 (100 %)	0.434	>0.999
High risk N1 (N=2)	1 (2.3 %)	1 (7.1 %)	0		
**In Age <45**	①Tumor ≤ 1.0 cm N=102 (68.0 %)	②1.0<Tumor ≤ 1.5 cm N=38 (25.3 %)	③1.5<Tumor ≤ 2.0 cm N=10 (6.7 %)	*p* value ①vs②	*p* value ②vs③
Low risk N1 (N=124)	87 (85.3 %)	29 (76.3 %)	8 (80.0 %)	0.217	>0.999
High risk N1 (N=26)	15 (14.7 %)	9 (23.7 %)	2 (20.0 %)		
**In Age ≥45**	①Tumor ≤ 1.0 cm N=91 (68.9 %)	②1.0<Tumor ≤ 1.5 cm N=32 (24.2 %)	③1.5<Tumor ≤ 2.0 cm N=9 (6.8 %)	*p* value ①vs②	*p* value ②vs③
Low risk N1 (N=123)	87 (95.6 %)	28 (87.5 %)	8 (88.9 %)	0.204	>0.999
High risk N1 (N=9)	4 (4.4 %)	4 (12.5 %)	1 (11.1 %)		

Data are presented as mean ± S.D. Pairwise Chi-square tests for discrete data, significance adjusted with Bonferroni correction ($p < 0.0167$ considered significant). Risk of recurrence of lymph node metastasis was classified as low risk (cN0, micro-metastasis, small LN metastasis, ≤5 small LN metastasis, and no extra-nodal extension) versus high risk (one or more of cN1, metastatic LN >3cm, >5 metastatic LNs, or extra-nodal extension).
