# Supplementary material for: Feasibility of active surveillance in patients with clinically T1b papillary thyroid carcinoma ≤1.5 cm in preoperative ultrasonography: MASTER study
Source: Eur Thyroid J. 2024 Apr 18;13(2):e230258. doi: 10.1530/ETJ-23-0258 (PMC11046321; doi:10.1530/ETJ-23-0258)
Supplement: Supplementary Table S5. Clinicopathologic factors associated with occult central LN metastasis in patients younger than 55 years [file supplementary_table_5.pdf]

Supplementary Table S5. Clinicopathologic factors associated with occult central LN metastasis in patients younger than 55 years

Parameters	Univariate regression		Multivariate regression	
	p value	Odds ratio (95% CI)	*p* value	Odds ratio (95% CI)
Age	<0.001	0.96 (0.94–0.98)	**<0.001**	**0.95 (0.93–0.98)**
Sex (ref.: female)	0.002	1.74 (1.22–2.49)	**0.041**	**1.66 (1.02–2.70)**
Tumor size	<0.001	3.12 (1.95–4.98)	**0.001**	**2.70 (1.47–4.96)**
Multifocality	0.740	1.07 (0.72–1.57)	0.271	1.32 (0.80–2.17)
Minimal ETE	<0.001	2.03 (1.47–2.82)	**0.001**	**2.06 (1.32–3.21)**
Vascular invasion	0.094	1.57 (0.93–2.66)	0.158	0.42 (0.13–1.39)
Lymphatic invasion	<0.001	4.26 (3.03–5.99)	**<0.001**	**3.26 (2.11–5.05)**
Thyroiditis	0.901	0.98 (0.68–1.41)	0.891	1.03 (0.64–1.68)
BRAF mutation	0.347	1.34 (0.73–2.49)	0.767	0.90 (0.44–1.82)

ETE, extrathyroidal extension; LN, lymph node. Univariate and multivariate logistic regression.
